# Underrepresentation of bats in Africa's protected areas

**DOI:** 10.1111/cobi.70108

**Published:** 2025-07-15

**Authors:** Cecilia Montauban, Ivana Budinski, Paul W. Webala, Theresa M. Laverty, Iroro Tanshi, Laura Torrent, Eric Bakwo‐Fils, Peter J. Taylor, Adam Kane, Ara Monadjem

**Affiliations:** ^1^ Department of Life Sciences Imperial College London Ascot UK; ^2^ Department of Life Sciences Natural History Museum London London UK; ^3^ Department of Genetic Research, Institute for Biological Research “Siniša Stanković” – National Institute of the Republic of Serbia University of Belgrade Belgrade Serbia; ^4^ Department of Forestry and Wildlife Management Maasai Mara University Narok Kenya; ^5^ Department of Fish, Wildlife and Conservation Ecology New Mexico State University Las Cruces New Mexico USA; ^6^ Department of Biology University of Washington Seattle Washington USA; ^7^ Small Mammal Conservation Organization Benin City Nigeria; ^8^ BiBio Research Group Natural Sciences Museum of Granollers Granollers Spain; ^9^ CIBIO‐InBIO, Research Centre in Biodiversity and Genetic Resources University of Porto Vairão Portugal; ^10^ Department of Environmental Sciences University of Ebolowa Ebolowa Cameroon; ^11^ Department of Zoology & Entomology and Afromontane Research Unit University of the Free State, Qwaqwa Campus Phuthaditjhaba South Africa; ^12^ Department of Biological Sciences University of Venda Thohoyandou South Africa; ^13^ Department of Biological Sciences University of KwaZulu‐Natal Durban South Africa; ^14^ School of Biology and Environmental Science and Earth Institute University College Dublin Dublin Ireland; ^15^ Department of Biological Sciences University of Eswatini Kwaluseni Eswatini; ^16^ Mammal Research Institute Department of Zoology & Entomology University of Pretoria Hatfield South Africa

**Keywords:** bat conservation, biodiversity, Chiroptera, conservation planning, parks, threatened species, biodiversidad, conservación de murciélagos, Chiroptera, especie amenazada, parques, planeación de la conservación, 蝙蝠保护, 生物多样性, 翼手目, 保护规划, 公园, 受威胁物种

## Abstract

Biodiversity is severely threatened globally, with habitat loss and other human pressures accelerating species extinctions. Protected areas (PAs) are a critical conservation tool; however, their effectiveness in safeguarding many taxa, such as bats, remains unclear. Using georeferenced occurrence records and species distribution models (SDMs) for 263 sub‐Saharan African bat species, we evaluated the coverage of bats in 7875 terrestrial PAs. Eighty‐nine percent of bat species were recorded in at least 1 PA, yet 28 species, including 5 threatened and 15 data deficient species, were absent from all PAs. Species with large extents of occurrence were represented in more PAs, and fruit bats occupied significantly more PAs than clutter, edge, or open‐air insectivorous foragers. The SDMs revealed high species richness in some undersurveyed areas, particularly in West and Central Africa and the Albertine Rift, emphasizing the need for targeted surveys. Our findings underscore critical data deficiencies related to bat conservation and stress the urgency of integrating bats into broader conservation planning. More surveys, enhanced data‐sharing, and tailored conservation strategies are needed to improve bat representation in PAs and safeguard their ecological roles in Africa's biodiverse landscapes.

## Abstract (es)

Subrepresentación de los murciélagos en las áreas protegidas de África

**Resumen:**

La biodiversidad sufre una amenaza grave en todo el mundo, y la pérdida de hábitats y otras presiones humanas aceleran la extinción de especies. Las áreas protegidas (AP) son una herramienta de conservación fundamental; sin embargo, su eficacia para proteger muchos taxones, como los murciélagos, sigue sin estar clara. Utilizamos registros georreferenciados de presencia y modelos de distribución de especies (MDE) de 263 especies de murciélagos del África subsahariana para evaluar la cobertura de murciélagos en 7,875 AP terrestres. El 89% de las especies de murciélagos se registraron en al menos una AP, sin embargo 28 especies, incluyendo 5 especies amenazadas y 15 especies con datos deficientes, estuvieron ausentes en todas las AP. Las especies con presencia extensa estuvieron representadas en más AP, y los murciélagos frugívoros ocuparon significativamente más AP que los insectívoros forrajeros de densidad vegetal, de borde o al aire libre. Los MDE revelaron una alta riqueza de especies en algunas áreas poco estudiadas, particularmente en África Occidental y Central y en la falla Albertina, lo que resalta la necesidad de estudios específicos. Nuestros hallazgos destacan las graves deficiencias de los datos relacionados con la conservación de los murciélagos y enfatizan la urgencia de integrar a los murciélagos en una planificación de conservación más amplia. Para mejorar la representación de los murciélagos en las áreas protegidas y salvaguardar su papel ecológico en la biodiversidad de los paisajes africanos se necesitan más estudios, un mejor intercambio de datos y estrategias de conservación adaptadas.

## 【摘要】

全球生物多样性正面临严重威胁, 栖息地丧失和其他人类活动正在加速物种灭绝。保护地是重要保护工具, 然而, 其在许多类群(如蝙蝠)保护方面的有效性仍不清楚。本研究利用263种撒哈拉以南非洲蝙蝠的地理参考出现记录和物种分布模型, 评估了7875个陆地保护地对蝙蝠的覆盖情况。结果表明, 89%的蝙蝠物种在至少1个保护地中有记录, 但有28种蝙蝠(包括5种受威胁物种和15种数据不足物种)在所有保护地中均未被记录。出现范围较广的物种在更多保护地中有分布记录, 而果蝠在保护地中的分布显著多余在杂乱环境、植被边缘或开放空间觅食的食虫性蝙蝠。物种分布模型显示, 一些调查不足的地区物种丰富度较高, 尤其是西非、中非和艾伯丁裂谷地区, 这强调了开展针对性调查的必要性。我们的研究结果突显了蝙蝠保护相关的关键数据空缺, 并强调了将蝙蝠纳入更广泛的保护规划的紧迫性。未来还应开展更多调查、加强数据共享, 以及制定针对性保护策略, 来提升蝙蝠在保护地中的代表性, 并保护其在非洲高生物多样性景观中的生态角色。**【翻译:胡怡思;审校:聂永刚】**

## INTRODUCTION

Biodiversity worldwide is under severe threat from habitat loss, degradation, pollution, and climate change (IPBES, 2019; Tittensor et al., 2014; Wolkovich et al., 2014). These human‐driven pressures, alongside many others, are putting countless species at risk, accelerating extinction rates, and undermining the stability and functioning of ecosystems globally (Cooke et al., 2023; Jantz et al., 2015; Pereira et al., 2024; Storch et al., 2022). Currently, over one fifth of known vertebrate species are threatened with extinction (Hoffmann et al., 2010; IUCN, 2024), including over a quarter of all mammal species (Cardillo et al., 2023; Sanders et al., 2024; Schipper et al., 2008). Africa, home to a vast array of unique ecosystems, is a refuge for numerous mammal species found nowhere else on Earth and plays a crucial role in preserving global biodiversity (Ceballos & Ehrlich, 2006; Turpie & Crowe, 1994; Visconti et al., 2011).

Protected areas (PAs) are a primary tool to mitigate biodiversity loss by safeguarding habitats from human impacts (Godet & Devictor, 2018). Globally, Target 3 of the Kunming–Montreal Global Biodiversity Framework—adopted by 196 countries in December 2022—aims to expand PAs to cover 30% of terrestrial, marine, and freshwater ecosystems by 2030 (CBD, 2022). However, economic pressures often undermine efforts to designate and manage PAs, leading to inadequate and uneven coverage across biomes and taxa (Oliveira et al., 2017; Symes et al., 2016; Watson et al., 2014). PAs in Africa cover over 4.3 million km^2^ of natural habitat (UNEP‐WCMC & IUCN, 2024), mostly renowned for their large, charismatic mammals (Craigie et al., 2010; Western et al., 2009). However, the efficacy of the African PA network has not yet been assessed at a continental scale for the majority of low‐profile and smaller‐bodied mammals (Rodrigues et al., 2004).

For many taxa, which species are present in PAs is yet to be determined, even though this is essential for conservation planning and is often used as a metric to evaluate PA effectiveness (Jennings, 2000; Van Breugel et al., 2015). Despite being the second‐most diverse group of mammals in the world, with 266 species recorded in sub‐Saharan Africa alone (Monadjem, Montauban, et al., 2024), bats (Mammalia: Chiroptera) are often excluded from biodiversity assessments, and significant gaps remain in understanding how well they are represented in PAs. At a local scale, bats are frequently the most species‐rich group of mammals in Africa, especially in savanna and forest habitats at elevations below 1500 m asl (Monadjem, Farooq, et al., 2024). Bats occupy a variety of habitats and can be broadly classified into 4 foraging assemblages based on their diet and foraging style: clutter, edge, and open‐air foragers (insectivorous species) and fruit bats (Monadjem, Farooq, et al., 2024). In addition to their diversity, bats deliver critical ecosystem services, such as pest control and seed dispersal (Aziz et al., 2021; Kunz et al., 2011; Tuneu‐Corral et al., 2023). Furthermore, they also exhibit varying levels of sensitivity to disturbance (Cleary et al., 2016; Kingston et al., 2003; Struebig et al., 2010), making them valuable bioindicators (Jones et al., 2009) that can provide nuanced insights into the effectiveness of PAs. However, bats remain poorly studied, and despite their ecological importance, they are rarely prioritized in conservation management or policy (Frick et al., 2020) and are frequently subjected to persecution (Kingston, 2016; Rocha et al., 2021).

Bats are poorly known partly because they are difficult to observe and identify without capture (Meyer et al., 2011; Tanshi et al., 2021) and have high levels of cryptic unresolved diversity (Baldwin et al., 2021; Demos et al., 2019; Monadjem, Guyton, et al., 2020; Patterson et al., 2024). Additionally, the difficulty of capturing different species varies considerably. Some bat families are frequently trapped across habitats (e.g., Pteropodidae), whereas others (e.g., Molossidae) forage above forest canopies and in open spaces, where they are difficult to catch or are more adept at avoiding capture (e.g., clutter foragers in the families Vespertilionidae, Rhinolophidae, and Hipposideridae) (Mande et al., 2023; Monadjem, Farooq, et al., 2024). These limitations result in incomplete occurrence records and poorly defined distributional ranges. Species distribution models (SDMs) help address these uncertainties by predicting bat distributions based on known occurrence points (Cooper‐Bohannon et al., 2016; Herkt et al., 2016; Razgour et al., 2016). Efforts to overcome this shortfall in knowledge of African bat distributions include the recently published African bat database (Monadjem, Montauban, et al., 2024)—a curated set of unique locality records of all bat species in sub‐Saharan Africa—and Maxent‐based SDMs.

We used georeferenced distributional data and models from the African bat database to evaluate the effectiveness of the existing PA network of mainland Africa in conserving sub‐Saharan bat species. In particular, we aimed to examine the coverage of species represented in PAs; identify species that have not been recorded in any PA; assess the coverage of species in PAs based on their family, extent of occurrence (EOO), extinction risk, and foraging assemblage; and compare whether using SDMs instead of actual occurrence records leads to different representation of bats in the PA network. We hypothesized that species with large geographic ranges intersect more PAs than species with small ranges, making range size the most important ecological correlate of the number of PAs in which a bat species occurs. We also hypothesized that due to their larger home range sizes (Wood et al., 2024), fruit bats and open‐air foragers are recorded more often in PAs than edge or clutter foragers and that threatened bat species are recorded in the fewest PAs due to their often‐restricted ranges and small population sizes.

## METHODS

### Data

We used bat species occurrence records, SDMs, and EOO (i.e., the area within the shortest continuous boundary that includes all known, inferred, or projected sites of a species’ current occurrence) from the African bat database (Monadjem, Montauban, et al., 2024). Maxent models were used by Monadjem, Montauban, et al. (2024) to develop SDMs for African bat species, run at a resolution of 2.5 arc minutes with BIOCLIM variables and elevation data from the WorldClim database (Hijmans et al., 2005). The Maxent models were run with the dismo R package (Hijmans et al., 2010), and detailed descriptions of the model testing and evaluation are in Monadjem, Montauban, et al. (2024).

We cropped records from the African bat database to mainland sub‐Saharan Africa only (excluding islands and north Africa above latitude 22°N). This retained 16907 unique locality records of 263 species (of the initial 266 species) from 12 families and 52 genera from sub‐Saharan Africa. Records covered all biomes and ranged in elevation from sea level up to 4207 m asl. We followed the taxonomy of Simmons and Cirranello (2025) and included the 16 taxa from the African bat database for which a specific species identity could not be assigned (i.e., species whose epithets begin with “cf.” followed by the name of the taxon closest in morphology). Maxent‐based SDMs were available for 208 species, and EOO was available for 238 species (Monadjem, Montauban, et al., 2024).

We compiled the conservation status of bats from the International Union for Conservation of Nature (IUCN) Red List (IUCN, 2024) and matched it to the most recent taxonomy with the Bat Taxonomy Alignment Tool (Sherman et al., 2023). Threatened species adhere to the IUCN definitions and were classified as vulnerable (VU), endangered (EN), and critically endangered (CR). Nonthreatened categories included least concern (LC) and near threatened (NT). Species listed as data deficient (DD) and not evaluated (NE) lack proper assessments of their extinction risk. We extracted the foraging assemblage of each bat species from Monadjem, Farooq, et al. (2024) and filled the assemblage of any missing taxa based on that of other species in the genus.

**FIGURE 1:**
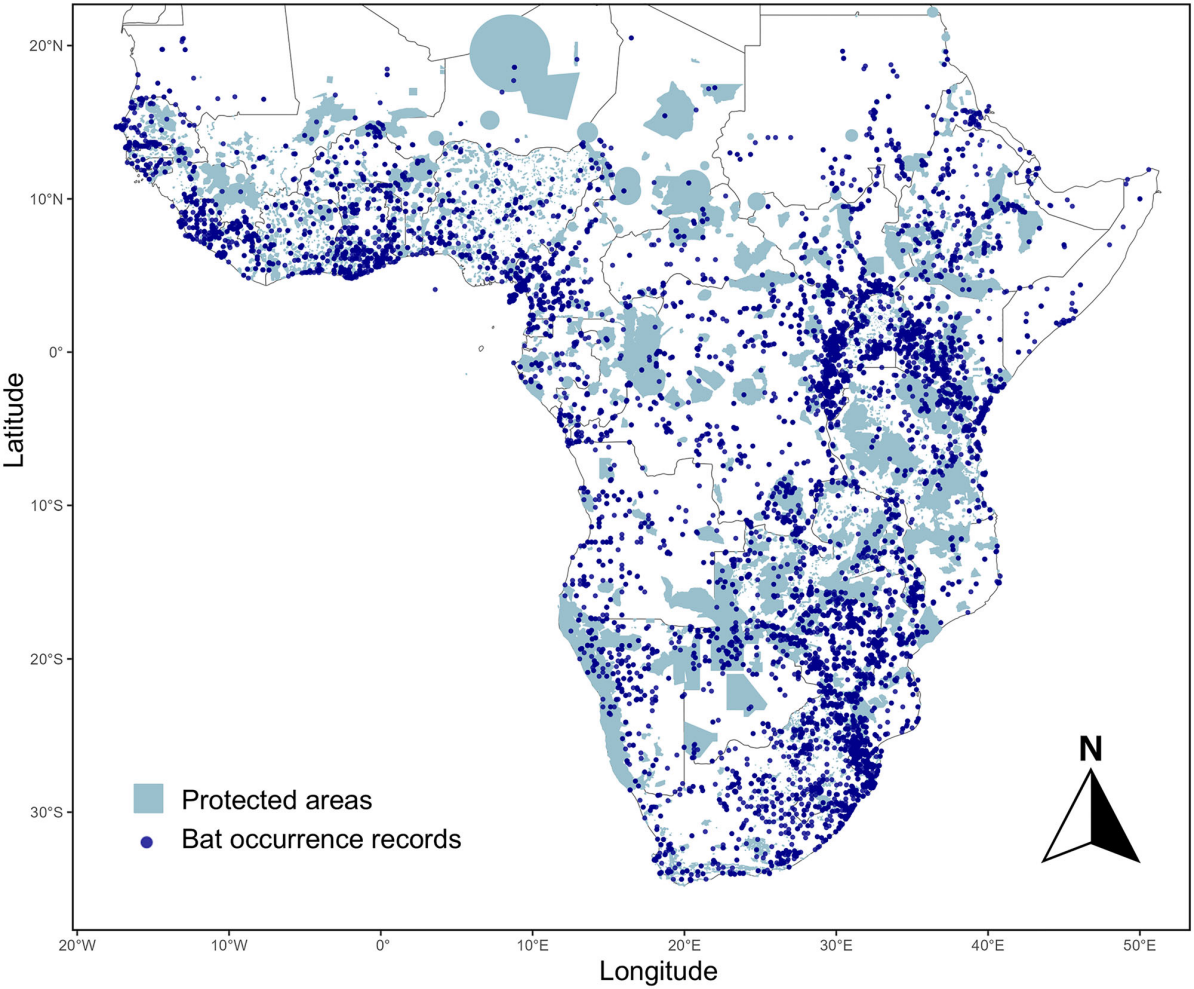
Sub‐Saharan African protected areas (*n* = 7875) and bat occurrence records (*n* = 16907) of the 263 sub‐Saharan bat species. Protected areas are from the December 2024 version of the World Database on Protected Areas layer (UNEP‐WCMC & IUCN, 2024), and occurrence records were extracted from the African bat database (Monadjem, Montauban, et al., 2024).

We downloaded shapefiles for PAs from the December 2024 version of the World Database of Protected Areas (WDPA) (UNEP‐WCMC & IUCN, 2024). The dataset includes PAs of all 7 IUCN PA management categories and all governance types—including federal PAs, privately owned areas, and Indigenous and local community‐based PAs. We filtered the dataset to retain only terrestrial PAs and incorporated both polygon and point data. Around 9% of WDPA spatial information was available only as point data (i.e., records lacking a defined boundary), and their inclusion enabled a more robust analysis when conducting a gap analysis for a given taxon (UNEP‐WCMC & IUCN, 2024). As recommended in the WDPA manual, we incorporated point data by creating a zone around the point with a radius proportional to the reported total area of the PA (UNEP‐WCMC & IUCN, 2024). We excluded point records for which area was not reported. All shapefiles were cropped to mainland sub‐Saharan Africa only (excluding islands and north Africa above latitude 22°N). The extent of the study area, PAs, and bat records are shown in Figure 1.

### Data analyses

All data analyses and mapping were done in R software (R Core Team, 2022). We used the overlay between occurrence records and PAs to calculate the number of PAs in which each species occurred and to identify bat species that were not recorded in any PAs. This was done by extracting occurrence records from the African bat database and overlaying them with the PA shapefile to extract records in PAs with the extract function in the terra R package (Hijmans et al., 2024).

To visualize the taxonomic coverage of bats in PAs, we plotted the number of PAs in which each species occurred on a phylogram with the ggtree R package (Yu et al., 2017). The backbone phylogenetic tree was adapted from Upham et al. (2019) with sequences of closely related species in the case of missing taxa and processed using the ape and phytools R packages (Paradis & Schliep, 2019; Revell, 2024). To assess differences in the representation of bat families in PAs, we compared the median number of PAs in which species of different bat families occurred with a Kruskal–Wallis test. This nonparametric test was selected because the data did not meet the assumptions of normality required for parametric alternatives.

We examined the coverage of bat species in PAs by modeling the total number of PAs each bat species occurred in (response variable) as a function of 3 explanatory variables: the species’ EOO (continuous variable), IUCN Red List category (7 categories), and foraging assemblage (4 categories). We fitted a generalized linear model with quasi‐Poisson distribution with the glm function from the stats R package (R Core Team, 2022). This model was selected to deal with overdispersion in the response variable count data. Model outputs were examined to assess the significance of each predictor in explaining variation in PA coverage among bat species.

To visualize the distribution and coverage of threatened (i.e., CR, EN, VU), DD, and NE bat species across sub‐Saharan Africa, we created a bivariate map showing conservation status in 2 categories (DD or NE species and threatened species), following an approach similar to that of Fernández‐Llamazares et al. (2021). We grouped PAs into 3 categories based on the number of bat species they contained. For threatened species, groups included PAs with 0 threatened species, 1–2 threatened species, and ≥3 threatened species. For the combined sum of DD and NE species, we grouped PAs into 3 categories: ≤2 DD–NE species, 3–5 DD–NE species, and ≥6 DD–NE species.

To determine whether using SDMs instead of actual occurrence records leads to different representation of bats in the PA network, we mapped species richness in PAs based on actual occurrence records and predicted records. The predicted species richness was calculated by overlaying the SDMs (instead of occurrence records) of each bat species with the PA shapefile to extract records in PAs.

## RESULTS

**FIGURE 2:**
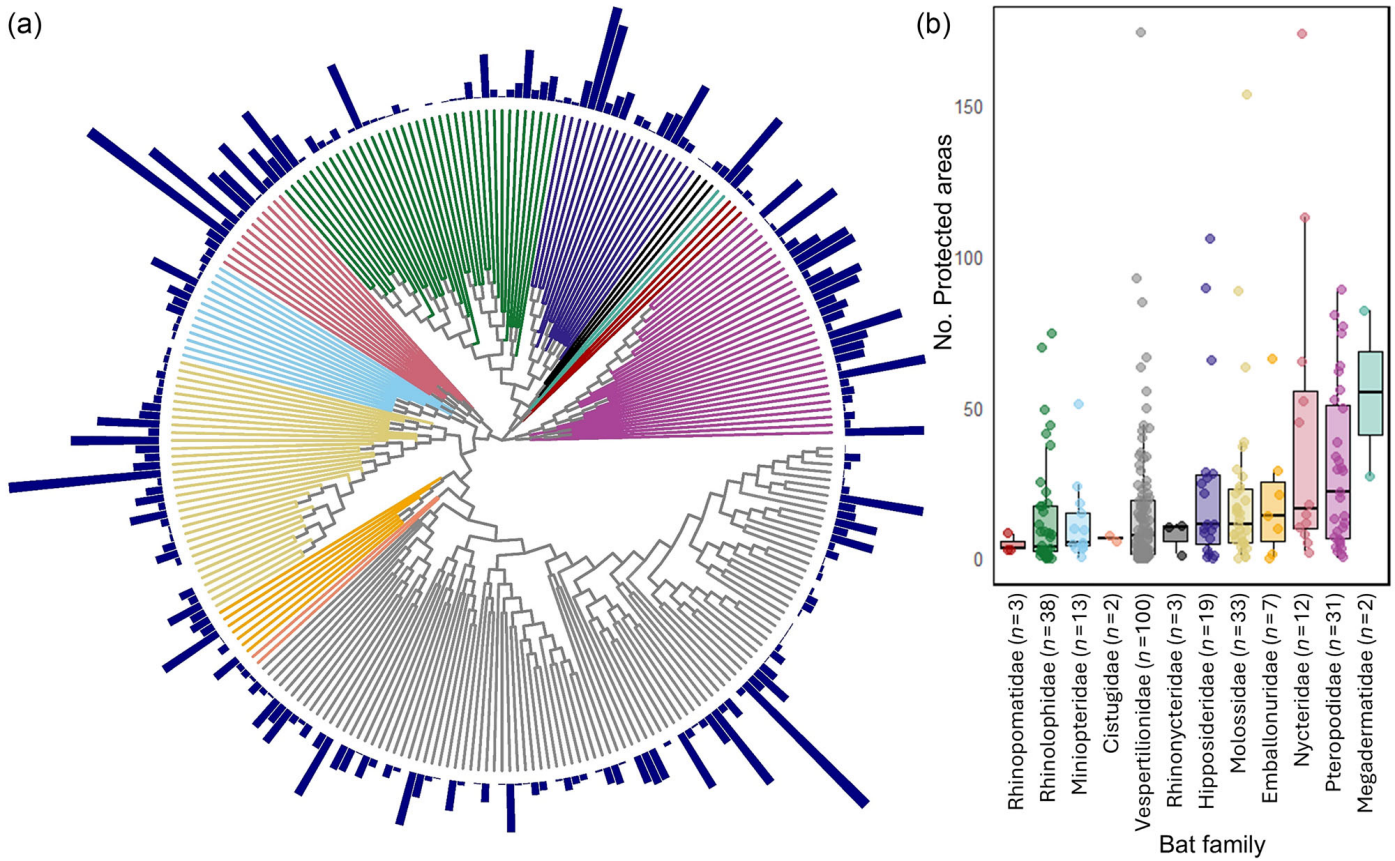
Taxonomic representation of bats in protected areas: (a) number of protected areas in which each of the 263 sub‐Saharan Africa bat species occurs (dark blue bars) mapped onto their phylogenetic tree (branches color‐coded by family) and (b) number of protected areas in which the species in each family occur.

A total of 16907 bat records were overlaid with 7875 PAs in mainland sub‐Saharan Africa (Figure 1). Bats were recorded in 855 of 7875 PAs (Appendix S1). There was a mean of 6.0 species per PA based on actual occurrence records. Based on actual occurrence records, 235 (89%) bat species were present in at least 1 PA. The number of PAs in which each bat species was recorded ranged from 0 to 174 (median = 9.0) (Figure 2a; Appendix S2). Twenty‐eight species from 7 families were not recorded in any PAs (Table 1), and 21 species occurred in just 1 PA. Three species stood out for being present in many PAs. *Afronycteris nana* (vespertilionid edge forager) and *Nycteris thebaica* (nycterid clutter forager) were both in 174 PAs. *Mops pumilus* (molossid open‐air forager) was in 154 PAs (Figure 2a,b; Appendix S2).

**TABLE 1:** The 28 species of sub‐Saharan African bats (*n* = 263) not recorded in any protected area.

Family	Species	IUCN Red List category	Foraging assemblage
Emballonuridae	*Taphozous hildegardeae*	VU	Open air
Hipposideridae	*Asellia italosomalica*	DD	Clutter
Hipposideridae	*Hipposideros megalotis*	LC	Clutter
Miniopteridae	*Miniopterus minor*	DD	Edge
Molossidae	*Mops gallagheri*	DD	Open air
Molossidae	*Mops niangarae*	DD	Open air
Molossidae	*Mops petersoni*	VU	Open air
Pteropodidae	*Epomophorus grandis*	DD	Fruit bat
Rhinolophidae	*Rhinolophus adami*	DD	Clutter
Rhinolophidae	*Rhinolophus mabuensis*	EN	Clutter
Rhinolophidae	*Rhinolophus sakejiensis*	DD	Clutter
Rhinolophidae	*Rhinolophus silvestris*	DD	Clutter
Rhinolophidae	*Rhinolophus willardi*	EN	Clutter
Vespertilionidae	*Barbastella leucomelas*	LC	Edge
Vespertilionidae	*Cnephaeus platyops*	DD	Edge
Vespertilionidae	*Glauconycteris atra*	NE	Edge
Vespertilionidae	*Glauconycteris kenyacola*	DD	Edge
Vespertilionidae	*Hypsugo ariel*	DD	Edge
Vespertilionidae	*Kerivoula eriophora*	DD	Clutter
Vespertilionidae	*Myotis dieteri*	DD	Edge
Vespertilionidae	*Myotis nimbaensis*	CR	Edge
Vespertilionidae	*Nycticeinops* cf. *grandidieri*	NE	Edge
Vespertilionidae	*Nycticeinops* cf. *macrocephalus*	NE	Edge
Vespertilionidae	*Pipistrellus kuhlii*	LC	Edge
Vespertilionidae	*Pipistrellus permixtus*	DD	Edge
Vespertilionidae	*Plecotus christii*	DD	Clutter
Vespertilionidae	*Scotophilus* cf. *andrewreborii*	NE	Edge
Vespertilionidae	*Scotophilus* cf. *dinganii*	NE	Edge

Abbreviations: CR, critically endangered; DD, data deficient; EN, endangered; IUCN, International Union for Conservation of Nature; LC, least concern; NE, not evaluated; NT, near threatened; VU, vulnerable.

The median number of PAs in which a species occurred was significantly different among bat families (χ^2^ = 57.93, df = 11, *p* < 0.001). It was lowest for Rhinopomatidae and Rhinolophidae (3.0 and 3.5 PAs, respectively) and highest for Pteropodidae and Megadermatidae (22.0 and 54.5 PAs, respectively) (Figure 2b).

**FIGURE 3:**
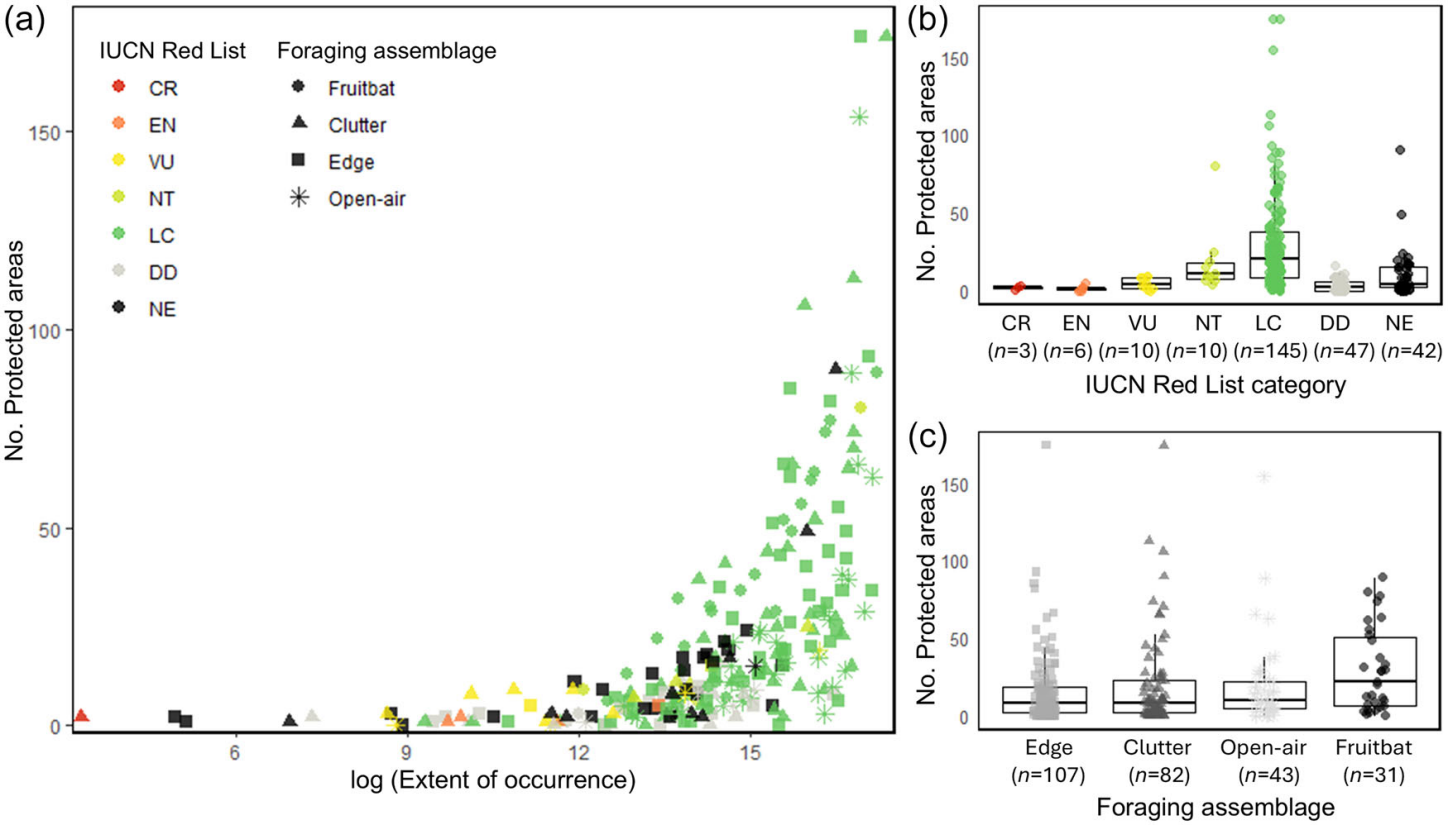
Relationship between extent of occurrence, International Union for Conservation of Nature (IUCN) Red List category and foraging assemblage of species, and their protected area coverage: (a) extent of occurrence (log‐transformed) relative to the number of protected areas each species occurs in (IUCN categories defined in Table 1), (b) number of protected areas in which the species in each IUCN Red List category occurs, and (c) number of protected areas in which the species in each foraging assemblage occur.

All explanatory variables had a significant effect on the number of PAs in which a species occurred: EOO (glm, *F* = 399.8, df = 1, *p* < 0.001), IUCN Red list category (glm, *F* = 2.4, df = 6, *p* < 0.05), and foraging assemblage (glm, *F* = 5.4, df = 3, *p* < 0.01). Generally, the larger the EOO of a species, the more PAs the species was recorded in (Figure 3a). Threatened bat species occurred in low numbers of PAs (Figure 3a,b). The number of PAs occupied by fruit bat species was significantly higher than that of species of other foraging assemblages, and it was higher for clutter foragers than for open‐air foragers (Figure 3c; Appendix S3).

**FIGURE 4:**
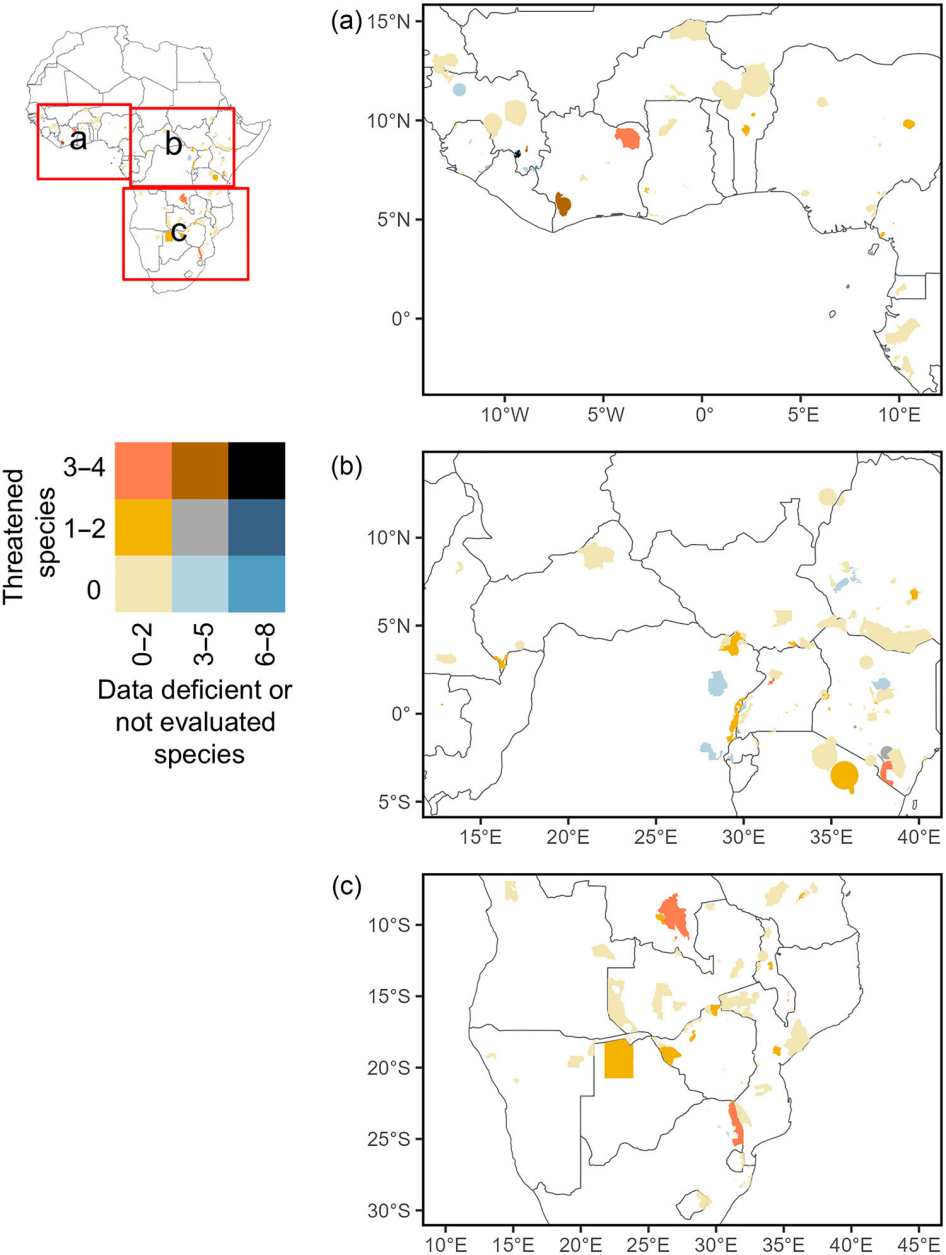
Protected areas harboring bat species classified as threatened, data deficient, and not evaluated under the International Union for Conservation of Nature Red List across 3 areas (a–c) in sub‐Saharan Africa and a bivariate map of the number of threatened (vulnerable, endangered, and critically endangered) bat species in protected areas versus the combined number of data‐deficient and not‐evaluated bat species in protected areas across sub‐Saharan Africa.

The distribution of threatened and DD–NE bat species in PAs based on actual occurrence records did not show any clustering in sub‐Saharan Africa (Figure 4). Massif du Ziama Forest (Guinea) and Mount Nimba (Guinea—Ivory Coast) had the most threatened and DD–NE bat species. Many other PAs had high numbers of DD–NE bat species, including Taï National Park and Comoé National Park (Ivory Coast), Kruger National Park (South Africa), Upemba National Park (Democratic Republic of the Congo), Tsavo West National Park (Kenya), and Budongo National Park (Uganda). The predicted PAs harboring higher numbers of threatened bats based on SDMs were in the upland regions of West Africa, especially the Liberia–Guinea border zone, tropical Cameroon, the Albertine Rift, and parts of Ethiopia, Kenya, Tanzania, and northeastern South Africa.

Of the 19 threatened bat species analyzed, 5 were not recorded in any PA, 3 were recorded in a single PA, and the rest were recorded in 2–9 PAs (Appendix S2). Of the 47 DD bats in sub‐Saharan Africa, 15 were not present in any PA and 7 were in just 1 PA (Appendix S2). The remainder of the DD species were present in 2–16 PAs. Finally, of the 42 NE species, 5 were not recorded in any PA, 4 were present in a single PA, and the rest occurred in 2–90 PAs (Appendix S2).

**FIGURE 5:**
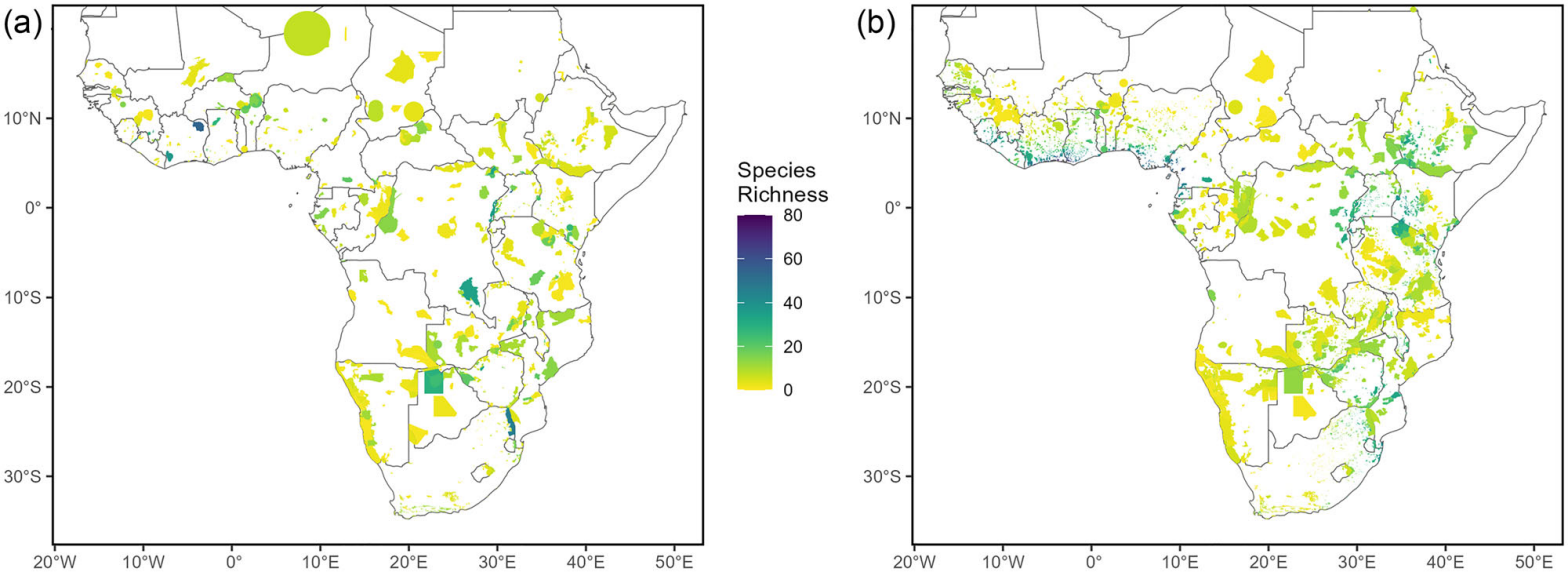
Number of bat species in protected areas in sub‐Saharan Africa based on (a) occurrence records and (b) species distribution models.

Species richness in PAs did not show any clear geographical pattern; species‐rich PAs were scattered throughout the continent (Figure 5a). Comoé National Park (Ivory Coast), Budongo National Park (Uganda), and Greater Kruger National Park (South Africa) had the highest bat species richness (54, 51, and 46 species, respectively). In contrast, the species richness of PAs based on the Maxent‐based SDMs predicted high species richness in specific regions of Central and West Africa, the Albertine and Gregory Rifts in East Africa, and central Mozambique (Figure 5b). The comparative number of bat species in PAs based on SDMs was far greater. Only 4 of the 208 species (1.9%) were predicted to not occur in PAs, and 35 species were predicted to occur in 1000 or more PAs (Appendix S2). The discrepancy between these 2 maps in Figure 5 is not surprising considering the large number of PAs that have yet to be surveyed for bats (Appendix S1).

## DISCUSSION

Our work is the first spatial analysis of the conservation status of sub‐Saharan African bats based on curated bat records. Our results highlight that, although 89% of bat species occur in at least 1 PA, the distribution of species is uneven across PAs. Some species were absent or poorly represented. Five threatened species, 15 DD species, and 5 NE species were absent from all PAs, underscoring critical gaps in conservation coverage. Moreover, the discrepancies between actual and predicted richness underscore the urgent need for comprehensive surveys to better inform conservation priorities.

The absence of 28 species from the PA network can be explained by multiple factors. Many species (e.g., *Pipistrellus permixtus*, *Glauconycteris kenyacola*, and *Mops gallagheri*) are only known from a type locality (Van Cakenberghe & Seamark, 2023) that is not in a PA. These species are so poorly known that nothing further can be said about their distributional ranges, let alone why they are absent from PAs. Some species, such as *P. permixtus*, also remain taxonomic mysteries; their phylogenetic relationships with congenerics are unclear (Monadjem et al., 2021). Others, such as *Miniopterus nimbae*, *Myotis nimbaensis*, *Rhinolophus namuli*, and *Pseudoromicia mbamminkom*, have been described only recently (Curran et al., 2022; Grunwald et al., 2023; Monadjem et al., 2019; Simmons et al., 2021) and need more sampling to determine their full distributional ranges. For at least a few of the 28 species, we suspect that their habitat does not occur in any PAs. This is particularly obvious for the arid zone of the Horn of Africa, where PA locations are not included in the WDPA but where at least 2 species that are not found in PAs (i.e., *Hipposideros megalotis* and *Asellia italosomalica*) are known to occur.

Species with large EOOs were recorded in more PAs, supporting our prediction that broader distributions lead to higher PA representation. This is in line with results of other studies (e.g., Pessoa Da Silva & De Marco Júnior, 2023). The geographic range of a species is part of the IUCN Red List criteria, which include whether the EOO is based on few occurrence records or whether the population has fluctuated or declined (IUCN, 2024). Therefore, it is unsurprising that many threatened species have small EOOs or lack an EOO estimation altogether due to limited records. Nonetheless, the lack of overlap between their distributions and PAs could be detrimental for the conservation of these species, particularly because natural habitats are increasingly threatened (Frick et al., 2020).

Fruit bats (Pteropodidae) occurred in significantly more PAs than all insectivorous foraging assemblages. Most pteropodids have generalist feeding habits, strong dispersal capabilities, and large home ranges. These characteristics allow them to use resources in a broad range of habitats, including disturbed landscapes (Egert‐Berg et al., 2021; Meijaard et al., 2005; Wood et al., 2024). This, along with their ease of capture, large size, and high visibility when roosting in trees, even in urban areas, likely contributed to more occurrence records and therefore higher representation in PAs. The comparative underrepresentation of insectivorous bats in PAs raises concern; the lack of protection of these species jeopardizes their conservation and the vulnerability of the pest‐suppression services they provide (Tuneu‐Corral et al., 2023). Among insectivorous foraging assemblages, differences were not significant, except between clutter foragers and open‐air foragers, with the former being represented in more PAs. Because clutter species mostly forage in forest interior habitats, this may be explained by the amount of PAs that are forested and the difficulty in surveying open‐air foragers (García et al., 2024; Struebig et al., 2010).

Our prediction that threatened species are underrepresented in PAs was supported and is consistent with the results of similar studies (Beresford et al., 2011; Venter et al., 2014). Of the 19 sub‐Saharan bat species classified as threatened, almost half were present in only 1 PA or no PAs, whereas the rest were found in fewer than 10 PAs. That 22 of the 47 DD species were recorded in at most 1 PA is also a cause for concern, particularly because the majority of DD species may in fact be threatened (Borgelt et al., 2022). Predicted species richness from SDMs suggests many unsurveyed PAs may host additional bat species, including threatened and DD species. PAs predicted to host high species richness included those in the upper and lower Guinean forests, the Congolian rainforest, central Mozambique, and the equatorial range of Eastern Africa. Species in these areas are currently underrecorded, and attention is needed to accurately assess their extinction risk, particularly for those still lacking an initial IUCN Red List evaluation, as a stepping stone to ensuring their conservation.

Our results showed a clear discrepancy in species richness of PAs based on actual occurrence records and predicted presence from SDMs, likely due to uneven and biased sampling (Fisher‐Phelps et al., 2017; Tanshi & Kingston, 2021). As with other similar studies (e.g., Struebig et al., 2010), use of predicted distributions improved the overall representation of bat species in PAs compared with use of actual species records. Predicted richness patterns in PAs align with estimated continental richness patterns (Herkt et al., 2016) but reflect substantial gaps in survey coverage. Bat surveys are heavily biased toward southern Africa (Monadjem, Taylor, et al., 2020), with large areas of tropical Africa still undersampled (Monadjem, Montauban, et al., 2024). This bias is evident in maps of actual records. Bat diversity hotspots, such as Kruger National Park (Rautenbach, 1982) and Comoé National Park (Fahr & Kalko, 2011), reflected sites with extensive survey efforts, whereas PAs in the Albertine Rift such as Virunga National Park (DRC) were underrepresented in bat research. Some occurrence records may not appear in SDMs due to limitations in species distribution modeling, particularly for microendemics, small sample sizes, or inaccurate environmental covariates (Synes & Osborne, 2011; Van Proosdij et al., 2016; Wisz et al., 2008). Acknowledging these discrepancies and biases underscores the urgent need for targeted surveys in less well‐sampled regions to improve bat biodiversity assessments. However, there will also be a need to create new PAs to conserve some of the bat species absent from the current PA network.

Despite being the most extensive worldwide database on PAs, the WDPA is still a dynamic dataset that remains incomplete. Some countries, such as Somalia, have not contributed spatial information (UNEP‐WCMC & IUCN, 2024). The IUCN's assessments also face challenges for inconspicuous species, such as bats, for which limited knowledge of population trends, abundance, and distribution often results in underestimated extinction risks (Edgar, 2025). Research and conservation efforts are hampered by severe data deficiencies, particularly in regions where bat diversity is highest but resources for their study and protection remain scarce. Many regions and taxa remain severely undersampled or are hindered by unresolved taxonomies, highlighting the urgent need for more targeted surveys and the submission of data to collaborative efforts like the African bat database (Monadjem, Montauban, et al., 2024). Without fundamental knowledge of species distributions, the ability to implement effective conservation strategies is severely hampered.

### Conservation implications

Our findings highlight the urgent need to integrate bat conservation more explicitly into PA management and expansion strategies across Africa. The lack of foundational data and the underrepresentation of many bat species in PAs, coupled with increasing threats from habitat loss, climate change, and roost disturbance (Frick et al., 2020), underscore the necessity of evidence‐based conservation planning that considers bats alongside other biodiversity priorities (Berthinussen et al., 2019; Sutherland et al., 2019).

PAs are often assumed to be safe havens for wild animals, but for many species this relationship has not yet been clearly established. The management of many African PAs is tailored to large, charismatic megafauna, which tend to be the central focus of ecotourism (Monadjem & Unwin, 2023). The proliferation of large megafauna may not always go hand in hand with populations of smaller vertebrates (McCleery et al., 2018). Species coexistence can have complex effects that influence whether specific PAs are viable structures for bat conservation. For example, savanna regions where PAs support large elephant populations may be detrimental to bats (Fenton et al., 1998; Parker & Bernard, 2023), whereas megaherbivores at lower densities in PAs in desert ecosystems may increase bat species richness and activity (Laverty & Berger, 2022). In general, PAs are a valuable conservation tool because they protect roosting sites (such as caves, crevices, and trees) and help maintain the ecosystem services provided by the bat populations that inhabit them (Cooper‐Bohannon et al., 2016; Tuneu‐Corral et al., 2025).

Deforestation and habitat loss remain one of the most pressing threats to bat diversity across the continent, driven largely by the expansion of small‐scale croplands and commodity crops (e.g., cacao, oil palm, rubber, and cashew nuts) (Masolele et al., 2024; Tyukavina et al., 2018). Urbanization poses another major threat because it leads to rapid transformation of natural habitats (Liu et al., 2016). Although some bat species can adapt to anthropogenic landscapes, many lose critical roosting and foraging sites (Frick et al., 2020; Russo & Ancillotto, 2015). Many bat species rely on continuous tree cover and cannot persist in fragmented landscapes (Meyer et al., 2016). PAs with well‐managed buffer zones and corridors can help maintain connectivity and reduce the impacts of habitat loss, particularly if the requirements of diverse types of taxa are considered in PA designation and planning (Ducci et al., 2019). Specific measures could include strict protection of forest fragments and riparian zones, as well as the identification and enhancement of key bat foraging areas in PAs.

Roost disturbance is another major concern, particularly for cave‐roosting bat species, which face threats from tourism, mining, hunting, and guano harvesting (Tanalgo & Hughes, 2025; Tanalgo et al., 2022). Implementing seasonal access restrictions, formal protection for critical roost sites, and sustainable guano harvesting practices would reduce disturbance and improve conservation outcomes. Similarly, hunting pressure on fruit bats for bushmeat and traditional medicine remains widespread (Mickleburgh et al., 2009; Mildenstein et al., 2016; Tackett et al., 2022). Tanalgo et al. (2023) found that higher numbers of bat species are hunted in countries with low PA coverage, suggesting that strong PA enforcement is needed to prevent illegal harvesting in reserves.

Our study represents the first attempt to understand the representation of sub‐Saharan African bat species in PAs and underscores the need for targeted conservation actions. We emphasize the urgent need for robust species inventories as a key step in integrating bats into PA planning and management, particularly in areas where undersampling and taxonomic uncertainties are hindering understanding of bat distributions and the requirements needed to conserve them. Future efforts should focus on integrating bats into PA conservation policies, strengthening monitoring programs, and promoting data sharing to enhance conservation efforts across the continent.

## Supporting information



Additional supporting information may be found in the online version of the article at the publisher's website.
