# Supplementary material for: Underrepresentation of bats in Africa's protected areas
Source: Conserv Biol. 2025 Jul 15;40(1):e70108. doi: 10.1111/cobi.70108 (PMC12856786; doi:10.1111/cobi.70108)
Supplement: Supplementary file 1 — Additional supporting information may be found in the online version of the article at the publisher's website. [file COBI-40-e70108-s001.docx]

**Supplementary Information**


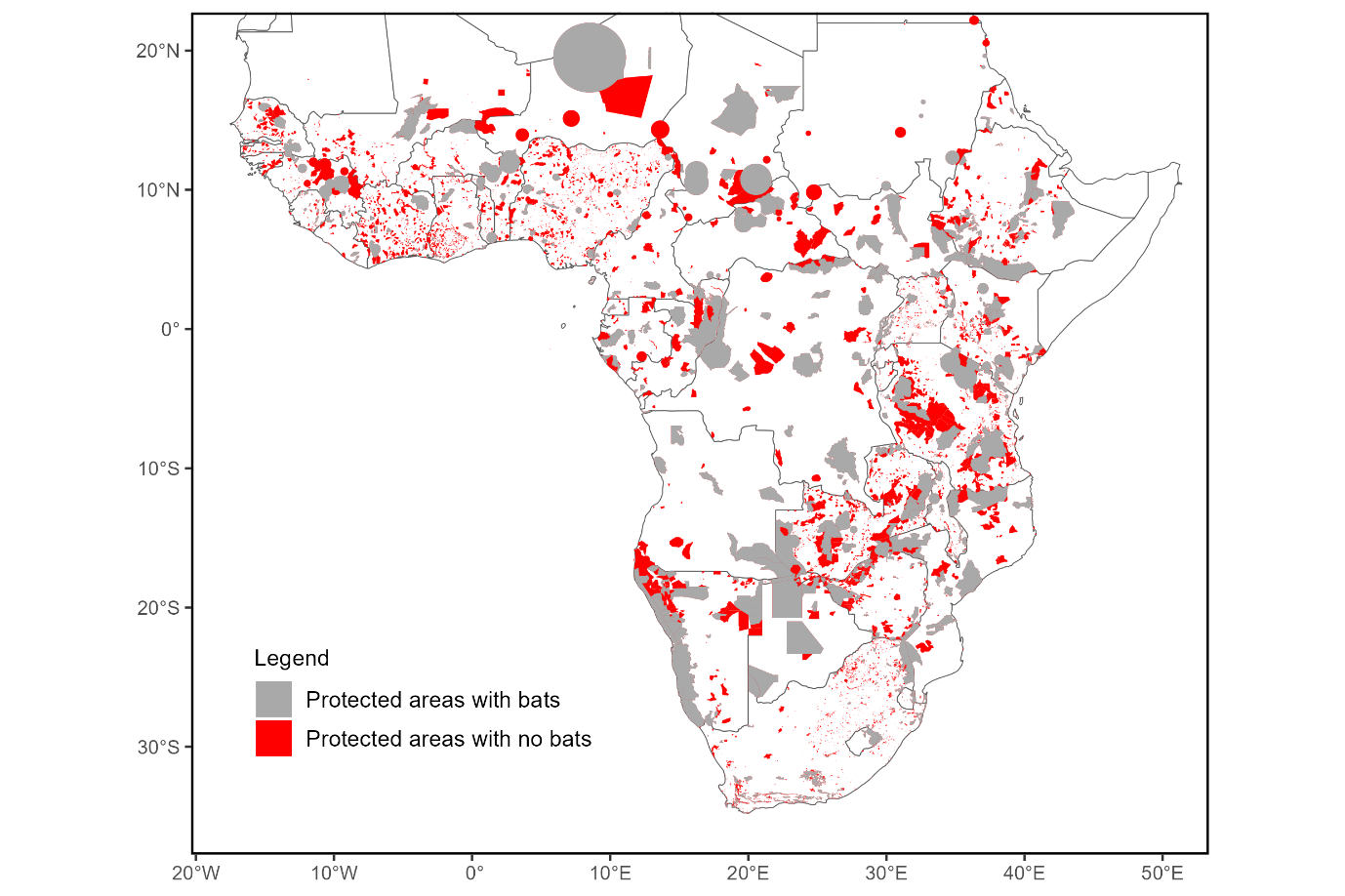


Appendix S1. Protected areas with (*n*= 855) and without (*n*= 7020) bat records, based on actual occurrence records.

Appendix S2. Summary of the number of protected areas (PAs) in which each of 263 bat species in sub-Saharan Africa are found, based on actual occurrence records and Maxent-based species distribution models (SDMs) from the African bat database (Monadjem, Montauban, et al. 2024). IUCN Red List threatened (vulnerable, VU; endangered EN; and critically endangered, CR), data deficient (DD) and not evaluated (NE) species are highlighted in different shades of blue. NA values indicate species for which SDMs were not available.

| **Family** | **Species** | **IUCN Red List Category** | **Foraging assemblage** | **No. of PAs (actual)** | **No. of PAs (SDMs)** |
| --- | --- | --- | --- | --- | --- |
| Cistugidae | *Cistugo lesueuri* | LC | Edge | 6 | 350 |
| Cistugidae | *Cistugo seabrae* | LC | Edge | 7 | 57 |
| Emballonuridae | *Coleura afra* | LC | Open-air | 14 | 728 |
| Emballonuridae | *Saccolaimus peli* | LC | Open-air | 21 | 317 |
| Emballonuridae | *Taphozous hamiltoni* | DD | Open-air | 1 | 1 |
| Emballonuridae | *Taphozous hildegardeae* | VU | Open-air | 0 | 14 |
| Emballonuridae | *Taphozous mauritianus* | LC | Open-air | 66 | 3040 |
| Emballonuridae | *Taphozous nudiventris* | LC | Open-air | 10 | 67 |
| Emballonuridae | *Taphozous perforatus* | LC | Open-air | 29 | 513 |
| Hipposideridae | *Asellia italosomalica* | DD | Clutter | 0 | NA |
| Hipposideridae | *Asellia patrizii* | LC | Clutter | 1 | NA |
| Hipposideridae | *Asellia tridens* | LC | Clutter | 9 | 218 |
| Hipposideridae | *Doryrhina camerunensis* | DD | Clutter | 11 | 83 |
| Hipposideridae | *Doryrhina cyclops* | LC | Clutter | 66 | 749 |
| Hipposideridae | *Hipposideros abae* | LC | Clutter | 11 | 721 |
| Hipposideridae | *Hipposideros beatus* | LC | Clutter | 28 | 507 |
| Hipposideridae | *Hipposideros caffer* | LC | Clutter | 106 | 1618 |
| Hipposideridae | *Hipposideros* cf. *ruber* | NE | Clutter | 90 | 912 |
| Hipposideridae | *Hipposideros curtus* | VU | Clutter | 1 | 222 |
| Hipposideridae | *Hipposideros fuliginosus* | LC | Clutter | 21 | 210 |
| Hipposideridae | *Hipposideros jonesi* | NT | Clutter | 10 | 164 |
| Hipposideridae | *Hipposideros lamottei* | CR | Clutter | 3 | NA |
| Hipposideridae | *Hipposideros marisae* | VU | Clutter | 8 | 10 |
| Hipposideridae | *Hipposideros megalotis* | LC | Clutter | 0 | 115 |
| Hipposideridae | *Hipposideros ruber* | LC | Clutter | 6 | 38 |
| Hipposideridae | *Hipposideros tephrus* | LC | Clutter | 27 | 1300 |
| Hipposideridae | *Macronycteris gigas* | LC | Clutter | 28 | 881 |
| Hipposideridae | *Macronycteris vittatus* | NT | Clutter | 25 | 1451 |
| Megadermatidae | *Cardioderma cor* | LC | Edge | 27 | 181 |
| Megadermatidae | *Lavia frons* | LC | Edge | 82 | 1154 |
| Miniopteridae | *Miniopterus africanus* | DD | Edge | 10 | 181 |
| Miniopteridae | *Miniopterus arenarius* | NE | Edge | 18 | 399 |
| Miniopteridae | *Miniopterus* cf. *fraterculus* | NE | Edge | 5 | 108 |
| Miniopteridae | *Miniopterus* cf. *inflatus* | NE | Edge | 15 | 172 |
| Miniopteridae | *Miniopterus* cf. *natalensis* | NE | Edge | 4 | 5 |
| Miniopteridae | *Miniopterus fraterculus* | LC | Edge | 3 | 184 |
| Miniopteridae | *Miniopterus inflatus* | LC | Edge | 5 | 60 |
| Miniopteridae | *Miniopterus minor* | DD | Edge | 0 | 370 |
| Miniopteridae | *Miniopterus mossambicus* | NE | Edge | 24 | 934 |
| Miniopteridae | *Miniopterus natalensis* | LC | Edge | 51 | 1005 |
| Miniopteridae | *Miniopterus nimbae* | NE | Edge | 3 | 0 |
| Miniopteridae | *Miniopterus villiersi* | NE | Edge | 9 | 54 |
| Miniopteridae | *Miniopterus wilsoni* | NE | Edge | 4 | 69 |
| Molossidae | *Mops aloysiisabaudiae* | LC | Open-air | 15 | 190 |
| Molossidae | *Mops ansorgei* | LC | Open-air | 27 | 676 |
| Molossidae | *Mops bemmeleni* | LC | Open-air | 7 | 1250 |
| Molossidae | *Mops bivittatus* | LC | Open-air | 24 | 969 |
| Molossidae | *Mops brachypterus* | LC | Open-air | 2 | NA |
| Molossidae | *Mops chapini* | LC | Open-air | 17 | 546 |
| Molossidae | *Mops condylurus* | LC | Open-air | 89 | 2345 |
| Molossidae | *Mops congicus* | LC | Open-air | 3 | 4 |
| Molossidae | *Mops demonstrator* | LC | Open-air | 13 | 71 |
| Molossidae | *Mops gallagheri* | DD | Open-air | 0 | NA |
| Molossidae | *Mops leonis* | NE | Open-air | 15 | 228 |
| Molossidae | *Mops major* | LC | Open-air | 29 | 555 |
| Molossidae | *Mops midas* | LC | Open-air | 37 | 355 |
| Molossidae | *Mops nanulus* | LC | Open-air | 16 | 384 |
| Molossidae | *Mops niangarae* | DD | Open-air | 0 | NA |
| Molossidae | *Mops nigeriae* | LC | Open-air | 38 | 342 |
| Molossidae | *Mops niveiventer* | LC | Open-air | 5 | 482 |
| Molossidae | *Mops petersoni* | VU | Open-air | 0 | NA |
| Molossidae | *Mops pumilus* | LC | Open-air | 154 | 2948 |
| Molossidae | *Mops russatus* | DD | Open-air | 4 | 270 |
| Molossidae | *Mops spurrelli* | LC | Open-air | 9 | 341 |
| Molossidae | *Mops thersites* | LC | Open-air | 23 | 462 |
| Molossidae | *Mops trevori* | DD | Open-air | 8 | 244 |
| Molossidae | *Myopterus daubentonii* | DD | Open-air | 5 | NA |
| Molossidae | *Myopterus whitleyi* | LC | Open-air | 6 | 272 |
| Molossidae | *Otomops harrisoni* | VU | Open-air | 8 | 1338 |
| Molossidae | *Otomops martiensseni* | NT | Open-air | 19 | 238 |
| Molossidae | *Platymops setiger* | LC | Open-air | 5 | 325 |
| Molossidae | *Sauromys petrophilus* | LC | Open-air | 21 | 122 |
| Molossidae | *Tadarida aegyptiaca* | LC | Open-air | 63 | 1073 |
| Molossidae | *Tadarida fulminans* | LC | Open-air | 11 | 354 |
| Molossidae | *Tadarida lobata* | LC | Open-air | 3 | 471 |
| Molossidae | *Tadarida ventralis* | DD | Open-air | 9 | 561 |
| Nycteridae | *Nycteris arge* | LC | Clutter | 45 | 211 |
| Nycteridae | *Nycteris aurita* | LC | Clutter | 5 | 391 |
| Nycteridae | *Nycteris gambiensis* | LC | Clutter | 12 | 617 |
| Nycteridae | *Nycteris grandis* | LC | Clutter | 52 | 489 |
| Nycteridae | *Nycteris hispida* | LC | Clutter | 113 | 1689 |
| Nycteridae | *Nycteris intermedia* | LC | Clutter | 14 | 50 |
| Nycteridae | *Nycteris macrotis* | LC | Clutter | 65 | 2333 |
| Nycteridae | *Nycteris major* | DD | Clutter | 8 | 314 |
| Nycteridae | *Nycteris nana* | LC | Clutter | 18 | 1229 |
| Nycteridae | *Nycteris parisii* | DD | Clutter | 2 | NA |
| Nycteridae | *Nycteris thebaica* | LC | Clutter | 174 | 2535 |
| Nycteridae | *Nycteris woodi* | LC | Clutter | 10 | 38 |
| Pteropodidae | *Casinycteris argynnis* | LC | Fruitbat | 11 | 13 |
| Pteropodidae | *Casinycteris campomaanensis* | DD | Fruitbat | 2 | NA |
| Pteropodidae | *Casinycteris ophiodon* | NT | Fruitbat | 6 | 15 |
| Pteropodidae | *Eidolon helvum* | NT | Fruitbat | 80 | 1880 |
| Pteropodidae | *Epomophorus angolensis* | NT | Fruitbat | 4 | 75 |
| Pteropodidae | *Epomophorus anselli* | DD | Fruitbat | 3 | NA |
| Pteropodidae | *Epomophorus crypturus* | LC | Fruitbat | 34 | 314 |
| Pteropodidae | *Epomophorus dobsonii* | LC | Fruitbat | 7 | 236 |
| Pteropodidae | *Epomophorus gambianus* | LC | Fruitbat | 52 | 551 |
| Pteropodidae | *Epomophorus grandis* | DD | Fruitbat | 0 | NA |
| Pteropodidae | *Epomophorus intermedius* | DD | Fruitbat | 1 | NA |
| Pteropodidae | *Epomophorus labiatus* | LC | Fruitbat | 62 | 1188 |
| Pteropodidae | *Epomophorus minimus* | LC | Fruitbat | 14 | 236 |
| Pteropodidae | *Epomophorus pusillus* | LC | Fruitbat | 74 | 1799 |
| Pteropodidae | *Epomophorus wahlbergi* | LC | Fruitbat | 64 | 882 |
| Pteropodidae | *Epomops buettikoferi* | LC | Fruitbat | 20 | 267 |
| Pteropodidae | *Epomops franqueti* | LC | Fruitbat | 49 | 754 |
| Pteropodidae | *Hypsignathus monstrosus* | LC | Fruitbat | 56 | 598 |
| Pteropodidae | *Megaloglossus azagnyi* | LC | Fruitbat | 32 | 401 |
| Pteropodidae | *Megaloglossus woermanni* | LC | Fruitbat | 38 | 346 |
| Pteropodidae | *Myonycteris angolensis* | LC | Fruitbat | 77 | 292 |
| Pteropodidae | *Myonycteris leptodon* | LC | Fruitbat | 22 | 255 |
| Pteropodidae | *Myonycteris relicta* | LC | Fruitbat | 6 | 150 |
| Pteropodidae | *Myonycteris torquata* | LC | Fruitbat | 29 | 221 |
| Pteropodidae | *Nanonycteris veldkampii* | LC | Fruitbat | 29 | 977 |
| Pteropodidae | *Plerotes anchietae* | LC | Fruitbat | 4 | 218 |
| Pteropodidae | *Rousettus aegyptiacus* | LC | Fruitbat | 89 | 1818 |
| Pteropodidae | *Scotonycteris bergmansi* | LC | Fruitbat | 10 | 137 |
| Pteropodidae | *Scotonycteris occidentalis* | LC | Fruitbat | 13 | 197 |
| Pteropodidae | *Scotonycteris zenkeri* | NT | Fruitbat | 9 | 93 |
| Pteropodidae | *Stenonycteris lanosus* | LC | Fruitbat | 30 | 236 |
| Rhinolophidae | *Rhinolophus acrotis* | LC | Clutter | 70 | 1632 |
| Rhinolophidae | *Rhinolophus adami* | DD | Clutter | 0 | NA |
| Rhinolophidae | *Rhinolophus alcyone* | LC | Clutter | 25 | 207 |
| Rhinolophidae | *Rhinolophus alticolus* | NE | Clutter | 8 | 92 |
| Rhinolophidae | *Rhinolophus blasii* | LC | Clutter | 15 | 653 |
| Rhinolophidae | *Rhinolophus capensis* | LC | Clutter | 8 | 267 |
| Rhinolophidae | *Rhinolophus cervenyi* | NE | Clutter | 3 | 79 |
| Rhinolophidae | *Rhinolophus cf. darlingi* | NE | Clutter | 3 | NA |
| Rhinolophidae | *Rhinolophus cf. denti* | NE | Clutter | 2 | 9 |
| Rhinolophidae | *Rhinolophus cohenae* | VU | Clutter | 3 | 251 |
| Rhinolophidae | *Rhinolophus damarensis* | LC | Clutter | 4 | 72 |
| Rhinolophidae | *Rhinolophus darlingi* | LC | Clutter | 37 | 424 |
| Rhinolophidae | *Rhinolophus deckenii* | NT | Clutter | 11 | 562 |
| Rhinolophidae | *Rhinolophus denti* | LC | Clutter | 2 | 10 |
| Rhinolophidae | *Rhinolophus eloquens* | LC | Clutter | 22 | 299 |
| Rhinolophidae | *Rhinolophus fumigatus* | LC | Clutter | 74 | 1183 |
| Rhinolophidae | *Rhinolophus gorongosae* | NE | Clutter | 1 | NA |
| Rhinolophidae | *Rhinolophus guineensis* | VU | Clutter | 9 | 34 |
| Rhinolophidae | *Rhinolophus hildebrandtii* | LC | Clutter | 17 | 421 |
| Rhinolophidae | *Rhinolophus hilli* | CR | Clutter | 2 | NA |
| Rhinolophidae | *Rhinolophus hillorum* | VU | Clutter | 3 | 41 |
| Rhinolophidae | *Rhinolophus hipposideros* | LC | Clutter | 2 | 0 |
| Rhinolophidae | *Rhinolophus landeri* | LC | Clutter | 18 | 945 |
| Rhinolophidae | *Rhinolophus lobatus* | NE | Clutter | 49 | 2301 |
| Rhinolophidae | *Rhinolophus mabuensis* | EN | Clutter | 0 | NA |
| Rhinolophidae | *Rhinolophus maclaudi* | EN | Clutter | 1 | 12 |
| Rhinolophidae | *Rhinolophus maendeleo* | DD | Clutter | 1 | NA |
| Rhinolophidae | *Rhinolophus mossambicus* | LC | Clutter | 41 | 381 |
| Rhinolophidae | *Rhinolophus namuli* | NE | Clutter | 2 | NA |
| Rhinolophidae | *Rhinolophus rhodesiae* | NE | Clutter | 17 | 163 |
| Rhinolophidae | *Rhinolophus ruwenzorii* | VU | Clutter | 9 | 14 |
| Rhinolophidae | *Rhinolophus sakejiensis* | DD | Clutter | 0 | NA |
| Rhinolophidae | *Rhinolophus silvestris* | DD | Clutter | 0 | NA |
| Rhinolophidae | *Rhinolophus simulator* | LC | Clutter | 44 | 472 |
| Rhinolophidae | *Rhinolophus smithersi* | NT | Clutter | 7 | 494 |
| Rhinolophidae | *Rhinolophus swinnyi* | LC | Clutter | 1 | 71 |
| Rhinolophidae | *Rhinolophus willardi* | EN | Clutter | 0 | NA |
| Rhinolophidae | *Rhinolophus ziama* | EN | Clutter | 2 | NA |
| Rhinonycteridae | *Cloeotis percivali* | LC | Clutter | 11 | 348 |
| Rhinonycteridae | *Triaenops afer* | LC | Clutter | 10 | 752 |
| Rhinonycteridae | *Triaenops persicus* | LC | Clutter | 1 | NA |
| Rhinopomatidae | *Rhinopoma cystops* | LC | Open-air | 8 | 13 |
| Rhinopomatidae | *Rhinopoma macinnesi* | DD | Open-air | 3 | 4 |
| Rhinopomatidae | *Rhinopoma microphyllum* | LC | Open-air | 3 | 161 |
| Vespertilionidae | *Afronycteris helios* | DD | Edge | 7 | 6 |
| Vespertilionidae | *Afronycteris nana* | LC | Edge | 174 | 1363 |
| Vespertilionidae | *Barbastella leucomelas* | LC | Edge | 0 | 0 |
| Vespertilionidae | *Cnephaeus floweri* | LC | Edge | 1 | 1 |
| Vespertilionidae | *Cnephaeus hottentotus* | LC | Edge | 19 | 843 |
| Vespertilionidae | *Cnephaeus platyops* | DD | Edge | 0 | NA |
| Vespertilionidae | *Glauconycteris alboguttata* | LC | Edge | 8 | 94 |
| Vespertilionidae | *Glauconycteris argentata* | LC | Edge | 13 | 909 |
| Vespertilionidae | *Glauconycteris atra* | NE | Edge | 0 | NA |
| Vespertilionidae | *Glauconycteris beatrix* | LC | Edge | 13 | 71 |
| Vespertilionidae | *Glauconycteris* cf. *humeralis* | NE | Edge | 2 | NA |
| Vespertilionidae | *Glauconycteris curryae* | DD | Edge | 2 | 252 |
| Vespertilionidae | *Glauconycteris egeria* | DD | Edge | 6 | 112 |
| Vespertilionidae | *Glauconycteris gleni* | DD | Edge | 2 | NA |
| Vespertilionidae | *Glauconycteris humeralis* | DD | Edge | 9 | 24 |
| Vespertilionidae | *Glauconycteris kenyacola* | DD | Edge | 0 | NA |
| Vespertilionidae | *Glauconycteris machadoi* | DD | Edge | 1 | NA |
| Vespertilionidae | *Glauconycteris poensis* | LC | Edge | 17 | 231 |
| Vespertilionidae | *Glauconycteris superba* | LC | Edge | 1 | NA |
| Vespertilionidae | *Glauconycteris variegata* | LC | Edge | 49 | 1460 |
| Vespertilionidae | *Hypsugo ariel* | DD | Edge | 0 | NA |
| Vespertilionidae | *Kerivoula africana* | EN | Clutter | 1 | NA |
| Vespertilionidae | *Kerivoula argentata* | LC | Clutter | 24 | 195 |
| Vespertilionidae | *Kerivoula cuprosa* | DD | Clutter | 8 | 381 |
| Vespertilionidae | *Kerivoula eriophora* | DD | Clutter | 0 | NA |
| Vespertilionidae | *Kerivoula lanosa* | LC | Clutter | 23 | 965 |
| Vespertilionidae | *Kerivoula phalaena* | LC | Clutter | 13 | 117 |
| Vespertilionidae | *Kerivoula smithii* | LC | Clutter | 4 | 140 |
| Vespertilionidae | *Laephotis angolensis* | DD | Edge | 16 | 190 |
| Vespertilionidae | *Laephotis capensis* | LC | Edge | 85 | 1594 |
| Vespertilionidae | *Laephotis* cf. *botswanae* | LC | Edge | 1 | 228 |
| Vespertilionidae | *Laephotis* cf. *capensis* | NE | Edge | 8 | 401 |
| Vespertilionidae | *Laephotis* cf. *wintoni* | NE | Edge | 2 | NA |
| Vespertilionidae | *Laephotis kirinyaga* | NE | Edge | 19 | 640 |
| Vespertilionidae | *Laephotis namibensis* | LC | Edge | 1 | NA |
| Vespertilionidae | *Laephotis stanleyi* | NE | Edge | 17 | 245 |
| Vespertilionidae | *Laephotis wintoni* | LC | Edge | 7 | 601 |
| Vespertilionidae | *Mimetillus moloneyi* | LC | Edge | 29 | 266 |
| Vespertilionidae | *Myotis bocagii* | LC | Edge | 55 | 2091 |
| Vespertilionidae | *Myotis dieteri* | DD | Edge | 0 | NA |
| Vespertilionidae | *Myotis morrisi* | DD | Edge | 1 | NA |
| Vespertilionidae | *Myotis nimbaensis* | CR | Edge | 0 | NA |
| Vespertilionidae | *Myotis scotti* | VU | Edge | 5 | 4 |
| Vespertilionidae | *Myotis tricolor* | LC | Edge | 31 | 802 |
| Vespertilionidae | *Myotis welwitschii* | LC | Edge | 24 | 1006 |
| Vespertilionidae | *Neoromicia anchietae* | LC | Edge | 6 | 118 |
| Vespertilionidae | *Neoromicia* cf. *anchietae* | NE | Edge | 1 | NA |
| Vespertilionidae | *Neoromicia guineensis* | LC | Edge | 15 | 757 |
| Vespertilionidae | *Neoromicia hlandzeni* | NE | Edge | 13 | 99 |
| Vespertilionidae | *Neoromicia somalica* | LC | Edge | 44 | 1189 |
| Vespertilionidae | *Neoromicia zuluensis* | LC | Edge | 43 | 650 |
| Vespertilionidae | *Nycticeinops bellieri* | NE | Edge | 11 | 43 |
| Vespertilionidae | *Nycticeinops* cf. *grandidieri* | NE | Edge | 0 | NA |
| Vespertilionidae | *Nycticeinops* cf. *macrocephalus* | NE | Edge | 0 | NA |
| Vespertilionidae | *Nycticeinops crassulus* | LC | Edge | 11 | 756 |
| Vespertilionidae | *Nycticeinops eisentrauti* | DD | Edge | 1 | NA |
| Vespertilionidae | *Nycticeinops grandidieri* | DD | Edge | 5 | 383 |
| Vespertilionidae | *Nycticeinops happoldorum* | NE | Edge | 3 | 1 |
| Vespertilionidae | *Nycticeinops macrocephalus* | NE | Edge | 2 | NA |
| Vespertilionidae | *Nycticeinops musciculus* | DD | Edge | 3 | 453 |
| Vespertilionidae | *Nycticeinops schlieffeni* | LC | Edge | 93 | 785 |
| Vespertilionidae | *Otonycteris hemprichii* | LC | Clutter | 4 | 0 |
| Vespertilionidae | *Pipistrellus aero* | DD | Edge | 4 | NA |
| Vespertilionidae | *Pipistrellus* cf. *hesperidus* | NE | Edge | 2 | NA |
| Vespertilionidae | *Pipistrellus hesperidus* | LC | Edge | 63 | 1267 |
| Vespertilionidae | *Pipistrellus inexspectatus* | DD | Edge | 8 | 2366 |
| Vespertilionidae | *Pipistrellus kuhlii* | LC | Edge | 0 | 36 |
| Vespertilionidae | *Pipistrellus nanulus* | LC | Edge | 33 | 60 |
| Vespertilionidae | *Pipistrellus permixtus* | DD | Edge | 0 | NA |
| Vespertilionidae | *Pipistrellus rusticus* | LC | Edge | 34 | 1357 |
| Vespertilionidae | *Pipistrellus simandouensis* | NE | Edge | 9 | 108 |
| Vespertilionidae | *Plecotus balensis* | DD | Clutter | 2 | NA |
| Vespertilionidae | *Plecotus christii* | DD | Clutter | 0 | 1 |
| Vespertilionidae | *Pseudoromicia brunnea* | NT | Edge | 15 | 28 |
| Vespertilionidae | *Pseudoromicia isabella* | DD | Edge | 1 | NA |
| Vespertilionidae | *Pseudoromicia kityoi* | NE | Edge | 1 | NA |
| Vespertilionidae | *Pseudoromicia mbamminkom* | NE | Edge | 1 | NA |
| Vespertilionidae | *Pseudoromicia nyanza* | NE | Edge | 17 | 585 |
| Vespertilionidae | *Pseudoromicia rendalli* | LC | Edge | 42 | 1557 |
| Vespertilionidae | *Pseudoromicia roseveari* | EN | Edge | 5 | 7 |
| Vespertilionidae | *Pseudoromicia tenuipinnis* | LC | Edge | 19 | 552 |
| Vespertilionidae | *Scotoecus albofuscus* | DD | Edge | 8 | 1771 |
| Vespertilionidae | *Scotoecus hindei* | LC | Edge | 21 | 1324 |
| Vespertilionidae | *Scotoecus hirundo* | LC | Edge | 20 | 329 |
| Vespertilionidae | *Scotophilus altilis* | NE | Edge | 2 | 202 |
| Vespertilionidae | *Scotophilus andrewreborii* | NE | Edge | 14 | 499 |
| Vespertilionidae | *Scotophilus* cf. *andrewreborii* | NE | Edge | 0 | NA |
| Vespertilionidae | *Scotophilus* cf. *dinganii* | NE | Edge | 0 | NA |
| Vespertilionidae | *Scotophilus damarensis* | NE | Edge | 21 | 168 |
| Vespertilionidae | *Scotophilus dinganii* | LC | Edge | 66 | 957 |
| Vespertilionidae | *Scotophilus ejetai* | LC | Edge | 9 | 10 |
| Vespertilionidae | *Scotophilus leucogaster* | LC | Edge | 40 | 588 |
| Vespertilionidae | *Scotophilus livingstonii* | LC | Edge | 26 | 703 |
| Vespertilionidae | *Scotophilus nigrita* | LC | Edge | 6 | 476 |
| Vespertilionidae | *Scotophilus nigritellus* | NE | Edge | 16 | 373 |
| Vespertilionidae | *Scotophilus nucella* | DD | Edge | 3 | NA |
| Vespertilionidae | *Scotophilus nux* | LC | Edge | 13 | 441 |
| Vespertilionidae | *Scotophilus trujilloi* | LC | Edge | 4 | 58 |
| Vespertilionidae | *Scotophilus viridis* | LC | Edge | 35 | 238 |
| Vespertilionidae | *Vansonia rueppellii* | LC | Edge | 34 | 1563 |


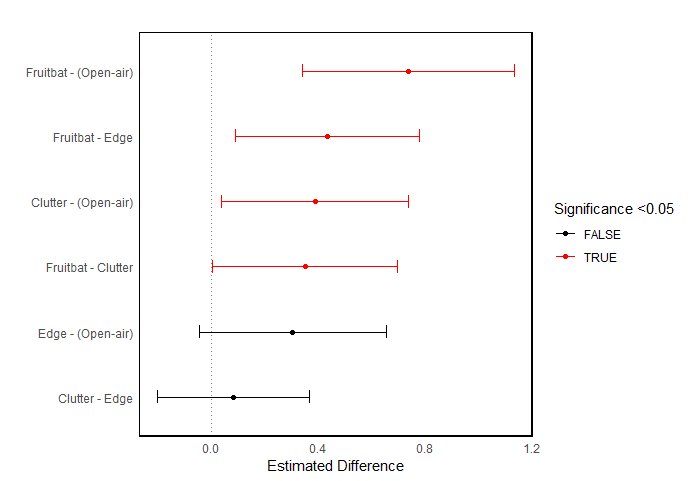


Appendix S3: Post-hoc Tukey pair-wise comparisons of the effect of a species’ foraging assemblage on the number of protected areas in which it occurs, Error bars indicate 95% confidence intervals.
